# Evaluation of the Effectiveness of Innovative Sorbents in Restoring Enzymatic Activity of Soil Contaminated with Bisphenol A (BPA)

**DOI:** 10.3390/molecules29133113

**Published:** 2024-06-29

**Authors:** Magdalena Zaborowska, Jadwiga Wyszkowska, Agata Borowik, Jan Kucharski

**Affiliations:** Department of Soil Science and Microbiology, University of Warmia and Mazury in Olsztyn, Plac Łódzki 3, 10-727 Olsztyn, Poland; m.zaborowska@uwm.edu.pl (M.Z.); agata.borowik@uwm.edu.pl (A.B.)

**Keywords:** BPA, remediating substances, adsorbents, *Zea mays*, enzymes activity

## Abstract

As part of the multifaceted strategies developed to shape the common environmental policy, considerable attention is now being paid to assessing the degree of environmental degradation in soil under xenobiotic pressure. Bisphenol A (BPA) has only been marginally investigated in this ecosystem context. Therefore, research was carried out to determine the biochemical properties of soils contaminated with BPA at two levels of contamination: 500 mg and 1000 mg BPA kg^−1^ d.m. of soil. Reliable biochemical indicators of soil changes, whose activity was determined in the pot experiment conducted, were used: dehydrogenases, catalase, urease, acid phosphatase, alkaline phosphatase, arylsulfatase, and *β*-glucosidase. Using the definition of soil health as the ability to promote plant growth, the influence of BPA on the growth and development of *Zea mays*, a plant used for energy production, was also tested. As well as the biomass of aerial parts and roots, the leaf greenness index (SPAD) of *Zea mays* was also assessed. A key aspect of the research was to identify those of the six remediating substances—molecular sieve, zeolite, sepiolite, starch, grass compost, and fermented bark—whose use could become common practice in both environmental protection and agriculture. Exposure to BPA revealed the highest sensitivity of dehydrogenases, urease, and acid phosphatase and the lowest sensitivity of alkaline phosphatase and catalase to this phenolic compound. The enzyme response generated a reduction in the biochemical fertility index (BA_21_) of 64% (500 mg BPA) and 70% (1000 mg BPA kg^−1^ d.m. of soil). The toxicity of BPA led to a drastic reduction in root biomass and consequently in the aerial parts of *Zea mays*. Compost and molecular sieve proved to be the most effective in mitigating the negative effect of the xenobiotic on the parameters discussed. The results obtained are the first research step in the search for further substances with bioremediation potential against both soil and plants under BPA pressure.

## 1. Introduction

Modern agricultural practices have played a significant role in increasing productivity in the 20th century. However, they have also led to environmental degradation in many parts of the world, as agriculture occupies about 38% of the world’s land area, totaling about 5 billion hectares [1]. Therefore, one of the greatest challenges is to meet the growing global demand for food and energy crops while taking into account the concept of a ‘circular economy’ [2]. This has led to an ‘ecological shift’ that is currently practiced in 187 countries and covers 72.3 million hectares of agricultural land [3]. These efforts are compounded and aligned with global industrialization, which, through industrial expansion, has contributed to a 65% increase in environmental pollution levels over the last 20 years [4]. Global market statistics indicate that plastics, including the production and use of plastic additives such as bisphenols, are most responsible for these trends [5,6].

It is not without reason that BPA has been commercially produced in the United States since 1957 [7]. Its potential lies in retarding the oxidative degradation of plastics exposed to UV radiation [8]. Desired are also its properties, such as high strength, heat resistance, and chemical stability [9], hence the impressive list of applications for BPA. Among the most important are the production of polycarbonates and associated electrical and electronic devices as well as building materials [10], epoxy resins, PVC, and polyesters [11]. Growing urbanization and changing lifestyle patterns contribute to the demand for plastic products, thereby driving the development of the BPA market. This trend reflects the expected compound annual growth rate of the global market for plastic additives, which is predicted to be 5.7% for the period 2021–2028 [12]. BPA production is also forecast to grow by 6% per year until 2027 [13].

Stringent government regulations on reclassifying BPA as a xenobiotic are emerging as a result of numerous scientific reports on its significant distribution in all ecosystems and, more alarmingly, its toxicity to organisms [14,15,16]. These regulations aim to limit the market expansion of BPA. One of the more significant regulations that came into force in 2023 is the acceptable daily intake of BPA at the level of 0.2 ng kg^−1^ body weight [17]. BPA has also been included in the Human Biomonitoring for Europe database, which defines the level of toxicity of this phenolic compound for humans [18], as well as in the Community Rolling Action Plan, a list of chemical compounds that are being evaluated for the risk they pose not only to human health but also to the environment [19]. These decisions are well founded, as the adverse effects of prolonged exposure to BPA have been linked to endocrine disruption [20], brain, and nervous system damage, and fetal development [21,22]. It is also a phenolic compound responsible for the induction of carcinogenic changes [23], insulin resistance, type 2 diabetes [24], and male infertility [25].

The dispersion of BPA in environmental media primarily refers to air, water, and soil. In the air, the main source of BPA is the thermal treatment of waste, mainly e-waste [26], as well as emissions resulting from its storage and transformation. In India, these practices have contributed to an increase in airborne BPA concentrations up to 4550 pg m^−3^ of air [27]. Currently, scientific discourse also considers the role of microplastics, which are thought to act as vectors for the transport of bisphenols in the air due to their adsorption by hydrogen and halogen bonds [28]. Leakages from landfills and discharges from wastewater treatment plants are the main sources of BPA pollution in aquatic environments. This issue is particularly prominent in rivers in Asia. In India, BPA levels of up to 14,800 ng dm^−3^ have been detected in river water [29]. In Europe, the highest levels of BPA in surface water have been found in Spain (28–560 ng dm^−3^ of water) [30].

Soil is exposed to BPA contamination from several undisputed sources. These include e-waste landfills [31], herbicide leaching [32], and treated wastewater used for irrigation [33]. Biosolids are also currently receiving considerable attention [34]. In Australia, their agricultural application increased to 73% in 2021, equivalent to 350,000 tons of biosolids [35]. In the European Union and the United States, it oscillates around 35% and 55%, respectively [36,37]. Staples et al. [38] documented the highest concentrations of BPA in European sediments (95,000 µg kg^−1^ d.m. of sediments) and much lower levels in North America (14,200 µg kg^−1^ d.m. of sediments).

The growing awareness of the persistent environmental pollution caused by xenobiotics has led to a wide range of global solutions to this problem. In order to maintain the philosophy of sustainable development, sorbents are increasingly being used for the remediation of contaminants in soils. This practice is supported not only by their affordable price but also by their market availability and, importantly, their cation exchange capacity [39]. Similarly, high-quality compost is receiving considerable attention from researchers, as it promotes the proliferation of microorganisms, thereby increasing the pool of enzymes [40,41,42]. Equally noteworthy as a soil fertilizing substance is coniferous bark, which constitutes over half of the 22 Mt of industrial bark produced annually in the European Union, particularly in Sweden and Finland [43]. Therefore, valuable applications for it are being sought, including in the production of ethanol, methane, and tannins, and as a substance with bioremediation properties [44,45].

An important component of the scientific debate on soil response to the pressure of organic pollutants is the analysis and consideration of soil enzyme responses. This variable, which describes soil quality, is recognized as a critical parameter and thus particularly reliable in assessing the extent of disturbance to its equilibrium resulting from the interaction of soil enzymes with phenolic compounds, including BPA [46,47]. These disturbances are also reflected in the response of cultivated plants. BPA is rapidly metabolized by a wide range of plant species, and the extent of disruption to their growth and development is closely related to the dose of this xenobiotic [48].

The current research objective was formulated taking into account observed trends related to the quest for answers to the question: (1) what is the potential scale of the inhibitory effect of BPA on soil condition? (2) It aimed to determine the response of seven soil enzymes to soil contamination with BPA, (3) expanded with the response of maize to the applied phenolic compound, and (4) to diagnose the bioremediation potential of (i) molecular sieve, (ii) zeolite SO1, (iii) sepiolite, (iv) starch, (v) grass compost, and (vi) fermented bark. Importantly, there have been no previous studies verifying the effectiveness of these sorbents against BPA pressure. The hypothesis was posited that this phenolic compound is responsible for significant soil equilibrium disturbance, reflected in both the reaction of soil enzymes and *Zea mays.* It was also assumed that the applied remediation substances would demonstrate varied potential in improving soil conditions, giving the research strictly practical significance.

## 2. Results

### 2.1. Enzymatic Activity of Soil

The results obtained from the biochemical analyses indicate that bisphenol A (BPA) is a potent inhibitor of soil enzymatic activity, with different responses observed between individual enzymes to this xenobiotic. This is demonstrated by the mean values obtained and their corresponding homogeneous groups in each treatment (Table 1). Considering the sensitivity of the seven enzymes analyzed to both 500 mg and 1000 mg BPA kg^−1^ of soil, as determined based on the mean values of these parameters, the following ranking can be proposed: dehydrogenases (Deh) > urease (Ure) > acid phosphatase (AcP) > *β*-glucosidase Glu > arylsulfatase (Aryl) > catalase (Cat) > alkaline phosphatase (AlP) (500 mg BPA) and Ure > Deh > AcP > Aryl > Glu > Cat > AlP (1000 mg BPA). The extent of BPA inhibition is also illustrated by the mean values of the soil fertility index (BA_21_), derived from the sum of all enzyme activities. It was observed that the application of 500 mg BPA kg^−1^ of soil resulted in a 64% lower BA_21_ value, and 1000 mg BPA kg^−1^ of soil resulted in a 70% decrease compared to the control. However, it should be emphasized that the activities of Deh, Ure, AcP, Aryl, and Glu were inhibited by BPA regardless of the soil contamination level with this phenolic compound. Conversely, an opposite trend was observed for AlP, and in the case of soil contaminated with 1000 mg BPA kg^−1^, Cat exhibited a different response. It was therefore essential to study the response of each enzyme separately in order to accurately describe the extent of the disturbance of the soil equilibrium assessed by its biochemical properties.

**Figure 1:**
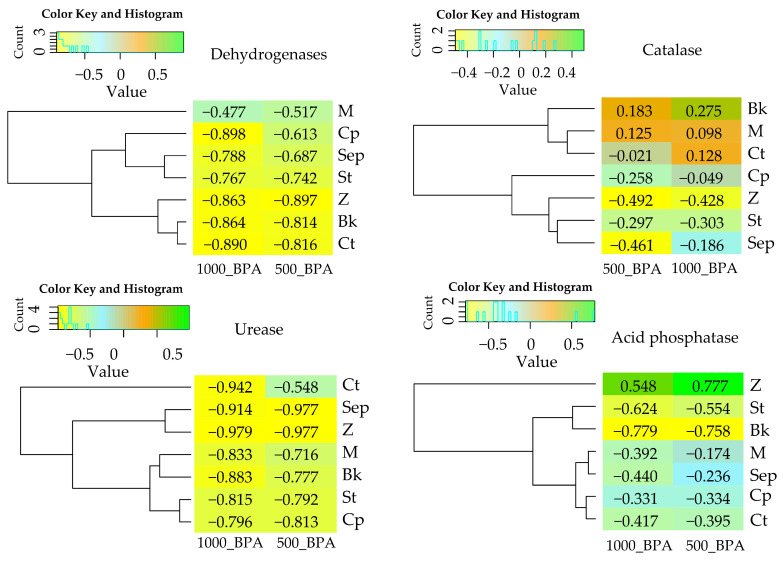

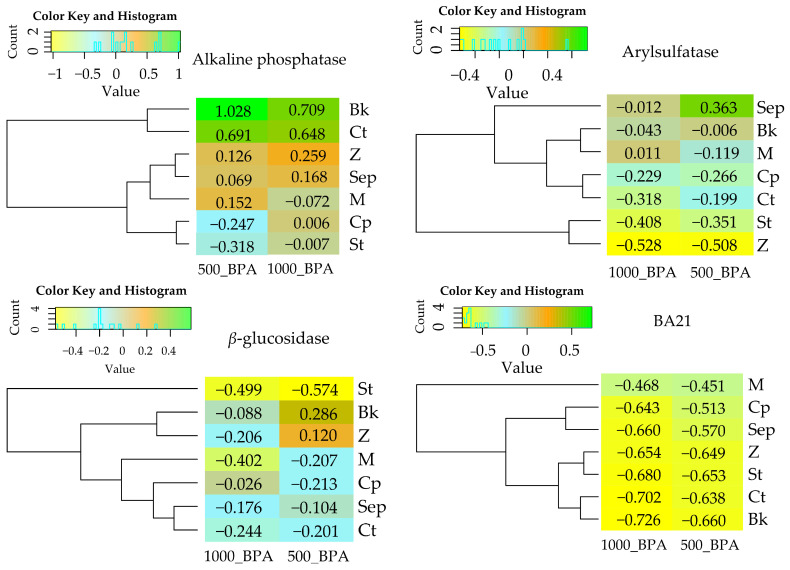
Index of the influence of BPA (IF_BPA_) on the activity of soil enzymes. Ct—soil without remediating substances, M—molecular sieve, Z—zeolite, Sep—sepiolite, St—Starch, Bk—pre-fermented bark, Cp—grass compost, 500_BPA, 1000_BPA—doses of BPA kg^−1^ d.m. of soil. The abbreviation IF_BPA_ is explained in the Section 4.

In uncontaminated soil, the activity of dehydrogenases was measured at 10.636 µmol TFF kg^−1^ of soil h^−1^ (Table 1). The application of 500 mg of the phenolic compound resulted in an 82% inhibition of the activity of these oxidoreductases, while 1000 mg resulted in 89% inhibition compared to the control. The values of the bisphenol influence factor (IF_BPA_) for the indicated treatments highlighted the trends observed for this parameter, assuming negative values of −0.816 and −0.890, respectively (Figure 1).

Studies indicate that catalase is less sensitive to bisphenol exposure than dehydrogenases. This is evidenced by the activity of this enzyme (Table 1), which increased by 13% at higher levels of BPA soil contamination (IF_BPA_ = 0.13) (Figure 1).

Within the hydrolase class, urease was the most sensitive enzyme to the influence of BPA. Importantly, in contrast to dehydrogenases, the effects of inhibition at 500 mg of this phenolic compound were not as drastic as at 1000 mg BPA kg^−1^ d.m. of soil. The pressure of individual doses of the xenobiotic resulted in a 55% and 94% inhibition of Ure activity, respectively, compared to the control objects. The trends observed corresponded to the negative values obtained for IF_BPA_ (−0.548 and −0.942) (Figure 1). The responses of acid phosphatase and alkaline phosphatase to BPA soil contamination were different. Importantly, the activity of AcP was observed to be 11-fold higher than that of AlP in soil not contaminated with the phenolic compound. However, the sensitivity of AcP to BPA action was disproportionately higher than that of AlP. Remarkably, regardless of the level of soil contamination with the xenobiotic, AcP activity was inhibited in the range of 40–42%, whereas AlP activity increased by 69% in response to 500 mg BPA and by 65% after the application of 1000 mg of the phenolic compound to the soil compared to the control.

The response of arylsulfatase and *β*-glucosidase to soil contamination with 500 mg of BPA was similar. In the sensitivity ranking of enzymes to this phenolic compound proposed above, Aryl and Glu ranked fourth and fifth, respectively. An escalation of Aryl inhibition was observed in soil exposed to 1000 mg BPA kg^−1^ d.m. of soil. This resulted in a 32% inhibition of enzyme activity and a negative IF_BPA_ index value of −0.318.

**Figure 2:**
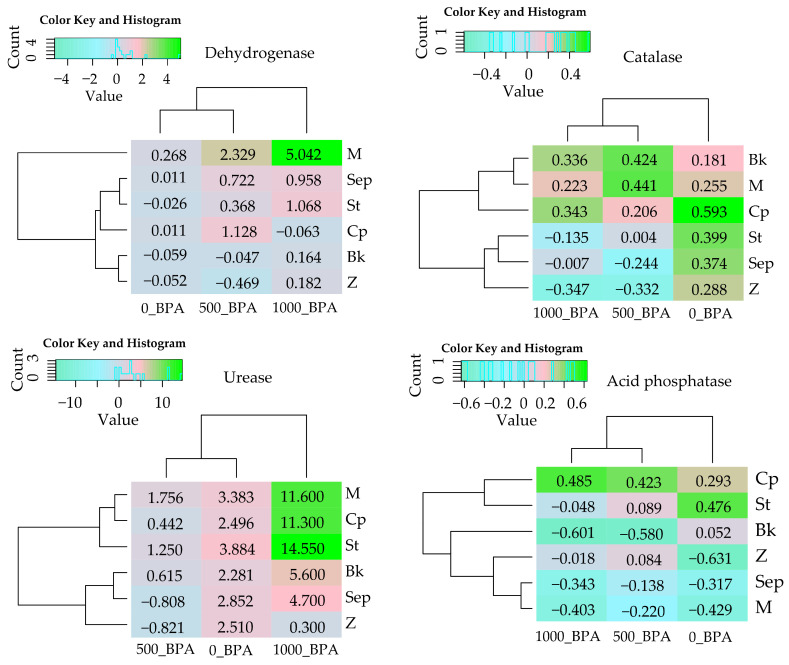

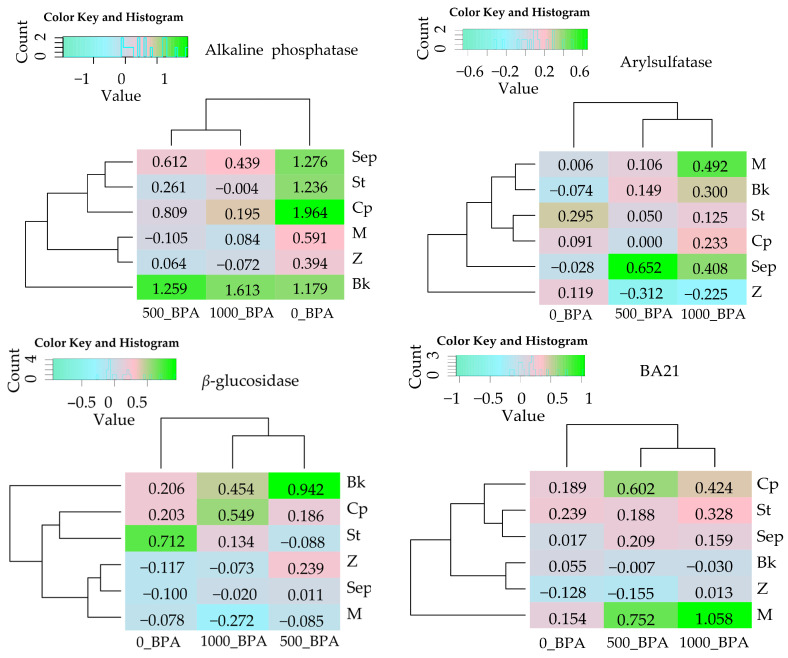
Index of the influence of remediation substances (IF_RS_) on the activity of soil enzymes. The abbreviations M, Z, Sep, St, Bk, and Cp are explained in Figure 1. The abbreviation IF_Rs_ is explained in the Section 4.

In pursuit of one of the defined research objectives, the potential of seven sorbents to restore the biochemical equilibrium of the soil was determined (Table 1, Figure 2). Mitigation of the negative effects of BPA on Deh was effective with the application of a molecular sieve (M). The adsorbent not only increased enzyme activity by 27% in uncontaminated soil but also induced a 233% increase in activity in soil exposed to 500 mg and a remarkable 504% increase in soil exposed to 1000 mg BPA kg^−1^ d.m. of soil. Positive influence indices of amendments on Deh activity also indicate the beneficial effects of grass compost (Cp) in soil exposed to 500 mg BPA (IF_RS_ = 1.128) and starch (St) in soil exposed to 1000 mg BPA kg^−1^ of soil (IF_RS_ = 1.068) (Figure 2). Although Cat proved to be a more resistant enzyme to the inhibitory effect of the phenolic compound, the applied sorbents did not play a significant role in stimulating its activity. The obtained positive IF_RS_ values for M, Co, and B in BPA-contaminated objects ranged only between 0.206 and 0.441. The effectiveness of the remediation substances, assessed on the basis of the urease reaction, confirmed the bioremediation potential of St, M, and Co, especially in soil contaminated with the highest dose of BPA. The urease activity increased from 0.020 mmol N-NH_4_ kg^−1^ of soil h^−1^ (Ct) to 0.351 (St), 0.252 (M), and 0.246 (Cp) mmol N-NH_4_ kg^−1^ of soil h^−1^ (Table 1), and the IF_SR_ values were 14.550 (St), 11.600 (M), and 11.300 (Cp), respectively (Figure 2). It is also worth noting that all applied substances had a positive effect on Ure activity. Based on the verification of changes in AcP and AlP activity, it was found that grass compost effectively mitigated the inhibitory effect of BPA on these enzymes. However, pre-fermented bark generated the highest increase in AlP activity, by 161% in soil contaminated with 500 mg BPA and by 126% after application of 1000 mg BPA kg^−1^ d.m. of soil.

The values of the influence indices (IF_RS_) on Aryl activity assigned to the objects with the compilation of BPA and individual sorbents indicated their relatively small positive effect on the soil condition, except for zeolite. Conversely, the assessment of their effectiveness through the prism of Glu reaction allowed for the identification of the bioremediation potential of pre-fermented bark. This was reflected both in the obtained average enzyme activity values, the assigned homogeneous groups, and the corresponding IF_RS_ values (0.942 and 0.454) in BPA-contaminated soil.

### 2.2. Zea mays

Given that soil condition defines its productivity, the response of *Zea mays* to BPA was monitored (Table 2). Considering the changes in the biomass of aerial parts and roots of the plants, a much stronger negative effect of higher doses of the xenobiotic on the growth and development of the cultivated plant was observed. The greater toxicity of BPA to its roots was also proven. The application of 500 mg BPA kg^−1^ soil resulted in a 60% inhibition of root yield, while the yield of aerial parts was reduced by 44% compared to the control. Conversely, in the group of objects subjected to the pressure of 1000 mg BPA kg^−1^ soil, the trend observed was a reduction in the yield of aerial parts of *Zea mays* similar to the yield of its roots. It oscillated at levels of 71% and 84%, respectively.

**Figure 3:**
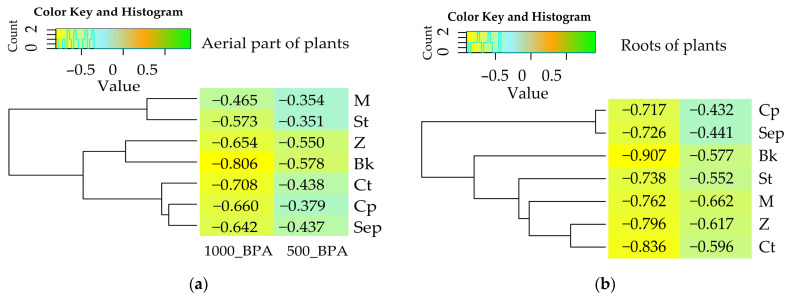
Index of the influence of BPA (IF_BPA_) on the yield of *Zea mays* (**a**) aerial parts of plants, (**b**) roots of plants. The abbreviations Ct, M, Z, Sep, St, Bk, and Cp are explained in Figure 1 and the abbreviation IF_BPA_ is explained in the Section 4.

The response of *Zea mays* to soil contamination with this phenolic compound, analyzed on the basis of mean yield values and their corresponding homogeneous groups, was reflected in the negative values obtained for the index of bisphenol A influence (IF_BPA_) in soil subjected to increasing levels of the xenobiotic (500 mg and 1000 mg). These values were significantly lower, particularly evident in the case of root biomass, with values of −0.596 and −0.836, respectively (Figure 3).

**Figure 4:**
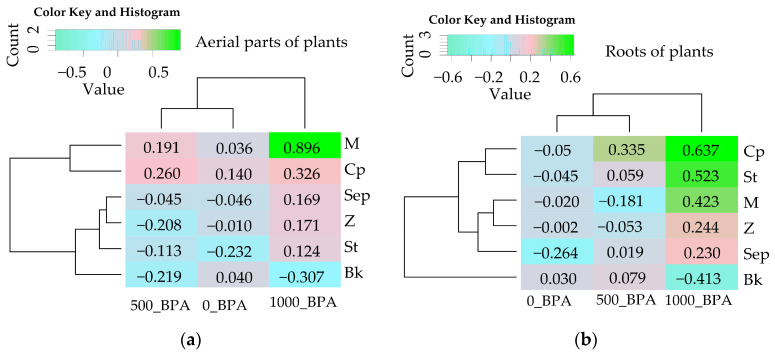
Index of the influence of remediation substances (IF_RS_) on the yield of *Zea mays* (**a**) aerial parts of plants, (**b**) roots of plants. The abbreviations M, Z, Sep, St, Bk, and Cp are explained in Figure 1 and the abbreviation IF_Rs_ is explained in the Section 4.

Soil contamination with BPA significantly disrupted the growth and development of *Zea mays* to such an extent that not all remediation substances applied were able to at least partially mitigate its adverse effects (Table 2, Figure 4). In the BPA-uncontaminated objects, the yield of aerial parts of *Zea mays* increased significantly after the application of compost, which was not observed in the case of plant roots. However, it is worth noting that in soil exposed to 500 mg kg^−1^ of soil, the bioremediation function was fulfilled by the molecular sieve (M) and compost (Cp). Their potential was further confirmed by the positive values of sorbent influence indices (IF_RS_) on the aerial parts of the plant, which were 0.191 and 0.260, respectively. In soil with a compilation of 1000 mg BPA and sorbents, only M and Cp were identified as being equally effective in promoting the growth and development of *Zea mays*. These compounds contributed to an increase in aerial part yield by 90% (M) and 33% (Cp) compared to the control, and in parallel objects, promoted root biomass growth of 42% (M) and 64% (Cp) (Figure 4). The observed trends were confirmed by positive IF_RS_ values ranging from 0.326 (Cp, aerial parts of plants) to 0.896 (M, aerial parts of plants), and from 0.423 (M, roots of plants) to 0.637 (Cp, roots of plants).

**Figure 5:**
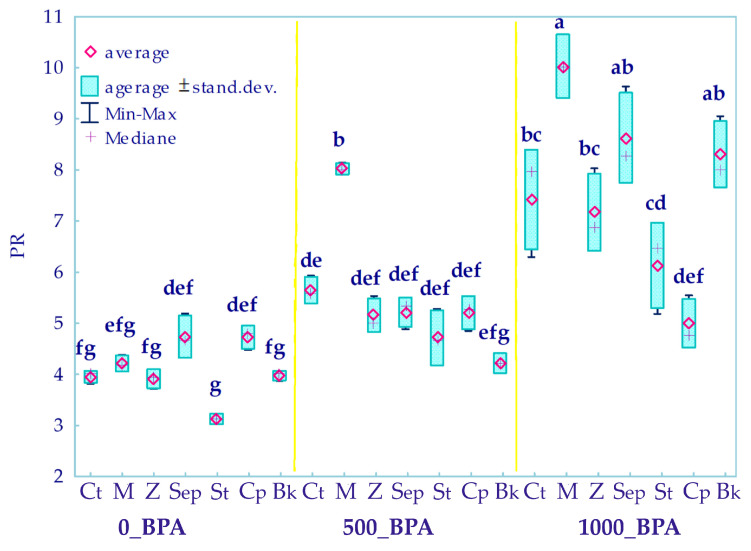
The ratio of the mass of the aerial parts of plants to the roots (PR) in the soil contaminated with BPA. The abbreviations are explained in Figure 1. Homogeneous groups were determined for the PR ratio assigned to all objects sown with *Zea mays* (represented by letters a–g), significant at *p* = 0.05, *n* = 63.

A validating parameter for the relationships described was the estimated ratio of aerial parts biomass to root biomass (PR) of *Zea mays* (Figure 5). Its value was significantly positively correlated with increasing soil contamination with BPA. The combination of the phenolic compound and the molecular sieve generated the highest PR values regardless of the magnitude of the xenobiotic level.

In the study conducted, the relative chlorophyll content was assessed using the SPAD leaf greenness index (Table 3). Increasing levels of soil contamination with BPA stimulated the synthesis of the photosynthetic pigment. The application of 500 mg BPA led to an increase in its content by 6%, and 1000 mg kg^−1^ of soil by 15%. The highest index values were recorded in soil subjected to the combinations of BPA and Cp, BPA and S, as well as in control objects enriched with Cp and Sep.

**Figure 6:**
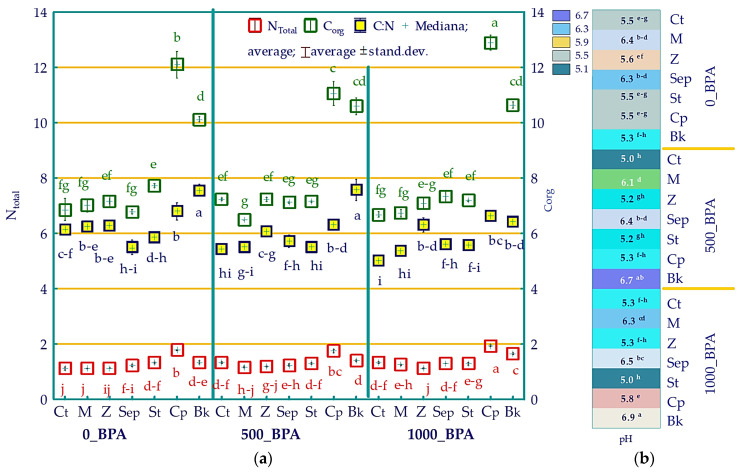
Total nitrogen content, the organic carbon content in g kg^−1^ d.m. of soil, and C:N ratio (**a**) and soil pH (**b**). Homogeneous groups were determined individually for N, C, C:N, and pH (represented by letters a–j (N_total_), a–g (C_org_), a–i (C:N), and a–h (pH). Abbreviations are explained in Figure 1, significant at *p* = 0.05, *n* = 63.

The phenolic compound introduced into the soil slightly moderated the nitrogen and organic carbon content, thereby affecting the soil C:N ratio (Figure 6a). The application of 500 mg BPA resulted in a 19% increase in nitrogen and a 5% increase in carbon ultimately leading to a decrease in the C:N value. Among the six remediating substances applied, Cp was responsible for a spectacular increase in soil organic carbon. It was twice as high in objects with soil exposed to 1000 mg BPA kg^−1^ of soil and in objects not contaminated with this xenobiotic compared to the control soil. Pre-fermented bark (Bk) was the second most effective in maintaining the trend described. Therefore, after its supplementation into the soil, the highest C:N ratio values were recorded in two sets of objects: Bk_0 = 7.561 and Bk_500 = 7.573, indicating a 23% increase in this parameter in each of them compared to the control. Regardless of the scale of soil contamination with BPA, molecular sieve and sepiolite induced an average increase in soil pH of one pH unit (Figure 6b). Pre-fermented bark also increased soil pH values, but only in objects with the combined effect of this remediation substance and the phenolic compound.

### 2.3. The Relationships between the Examined Properties: Percentage of Variability of the Analyzed Variable (η^2^) and PCA

**Figure 7:**
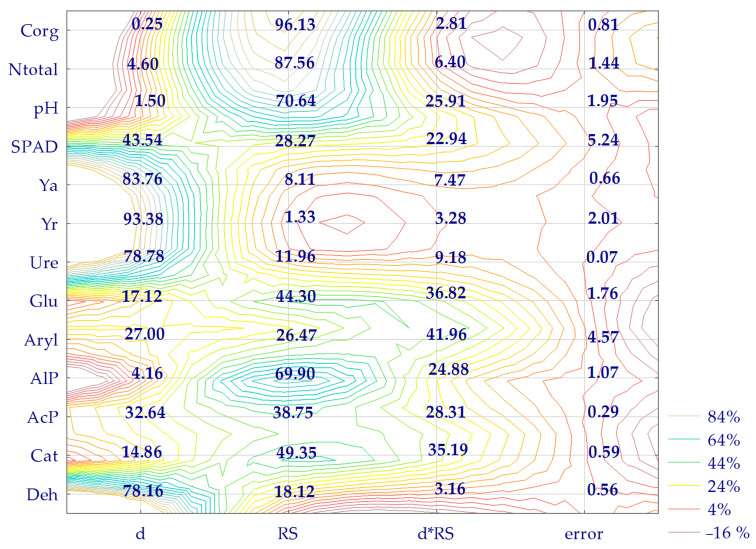
The share of independent variables (η^2^) in the change of soil enzyme activity, a yield of aerial parts and roots of *Zea mays*, SPAD, soil pH, total nitrogen, organic carbon, in %. d—dose of BPA kg^−1^ d.m. of soil, RS—remediation substance, Y_a_—aerial part of plants, Y_r_—roots of plants, N_total_—total nitrogen, C_org_—organic carbon, Deh—dehydrogenases, Cat—catalase, Ure—urease, AcP—acid phosphatase, AlP—alkaline phosphatase, Aryl -arylsulfatase, Glu—*β*-glucosidase.

All the dependent variables characterized in the studies were moderated to varying degrees by the independent variables defined in the experiment (Figure 7). Based on the obtained η^2^ values, it was estimated that biotic stress induced by soil contamination with BPA affected the response of *Zea mays* more than soil enzymes or changes in total nitrogen (N_total_), organic carbon (C_org_), and soil pH. Under increasing BPA pressure, the yield of the aerial parts of the cultivated plant and the roots underwent changes at levels of 84% and 93%, respectively. Analyzing the different enzyme responses to BPA in the soil revealed the following sequence: Ure (78%) > Deh (79%) > AcP (33%) > Aryl (27%) > Glu (17%) > Cat (15%) > AlP (4%). The remediation substances applied proved to be a much more significant factor in determining the biochemical activity of the soil. Considering the extent of their intervention, enzymes were ranked in the following order: AlP (70%) > Cat (49%) > Glu (44%) > AcP (38%) > Aryl (26%) > Deh (18%) > Ure (12%). Sorbent supplementation proved to be less significant for the growth and development of *Zea mays*. However, it strongly influenced the content of C_org_ (96%), N_total_ (88%), and soil pH (71%).

**Figure 8:**
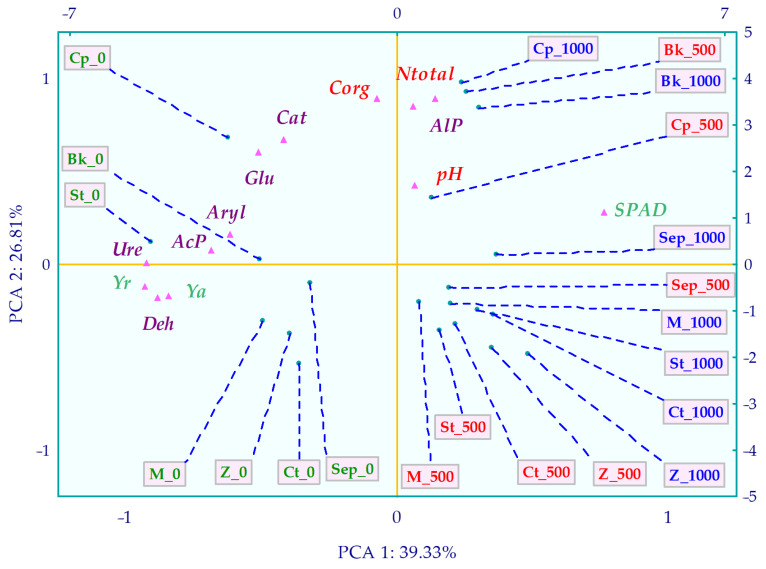
Interdependencies among all parameters characterized in the research—PCA method. Abbreviations are explained in Figure 1 and Figure 7.

The association of thirteen research parameters allowed the delineation of the relationships between them (Figure 8). These were explored using multivariate PCA. The PCA highlighted the extent of BPA interference and the bioremediation potential of the applied remediation substances. 39.33% of the total data variance, explained by the first principal component variable, was related to five soil enzymes: Deh, Ure, AcP, Aryl, Glu, and the SPAD index. The second variable, describing 26.81% of the variable variance (PCA2), illustrated the response of the Cat enzyme and AlP, parameters characterizing the response of *Zea mays*: Y_a_, Y_r_, as well as changes in soil N_total_, C_org_, and pH. The distribution of all cases demonstrated that BPA in the soil had an inhibitory effect on the six enzymes mentioned above. Their displacement further highlighted the beneficial effect of the sorbents applied to the soil on the biomass of *Zea mays* aerial parts. It also indicates a positive correlation between AlP activity and soil C_org_ and N_total_ contents, while emphasizing the importance of soil pH in this context.

## 3. Discussion

### 3.1. Soil Enzymes

The selection of soil enzymes as reliable indicators of soil condition was dictated by the fact that they play a key role in catalyzing the decomposition of organic matter by reducing the activation energy associated with these reactions [49]. The defined activity of seven soil enzymes in our study and the biochemical fertility index BA_21_ estimated from them accurately delineated the extent of the disturbance in soil equilibrium resulting from increasing bisphenol A (BPA) pressure on these parameters. Each of the soil enzymes analyzed shaped its value to a different extent. Deh, Ure, AcP, Aryl, and Glu were sensitive to BPA soil contamination.

Particularly sensitive to the tested xenobiotic were dehydrogenases, enzymes involved in the anabolic and catabolic pathways of living microorganisms, located in their polysomes and cytoplasm [50]. Scientific reports [51,52], which do not unequivocally indicate the toxicity of the tested phenolic compound to Deh, prompt a discussion on the reactions of these enzymes. In the study by Zaborowska et al. [51], Deh activity was found to be three times higher in soil contaminated with 100 mg BPA kg^−1^ d.m. of soil compared to control objects. It could be presumed to be the correct trend, considering that these are enzymes involved in the dehydrogenation of phenolic compounds. The hydrogen obtained in the bioutilization process of the organic substrate is transferred to the microbiological respiratory chain, ultimately benefiting the biosynthesis process and cellular metabolism [53]. Dehydrogenases also participate in the final degradation of acetophenone to benzaldehyde and benzoic acid [52]. However, to specify, the mechanism of stimulation was not necessarily based on an increase in the activity of Deh exposed to BPA, but rather on the accumulation of the enzyme by microorganisms, as a response to oxidative stress [54]. Moreover, in other studies, the application of 800 mg BPA kg^−1^ d.m. of soil [55] and 1000 mg BPA kg^−1^ d.m. of soil [56] resulted in inhibition of Deh activity by 21% and 50%, respectively, compared to the control. The most credible explanation for these correlations is the demonstrated toxicity of BPA biodegradation intermediates, mainly hydroquinone [57].

The inhibitory effect of BPA on urease exposed in our studies is confirmed by the results obtained by other researchers [34] and our previous reports [55,56]. They showed an inhibitory effect of the phenolic compound on urease at the level of 22% under the pressure of 800 mg BPA [55] and 13% below 100 mg BPA kg^−1^ dry soil [51] compared to uncontaminated soil. The positioning of the hydroxyl group and other important substituents, including N1 and N2-diaryl derivatives in the phenyl ring, played a significant role, as suggested by Mustafa et al. [58] and Perveen et al. [59]. More controversy was raised by the effect of BPA on phosphatase activity, particularly in inducing an increase in alkaline phosphatase (AlP) activity. Similar trends were observed in our previous studies [56], although the stimulation strength at 1000 mg BPA kg^−1^ d.m. of soil was higher than in the current studies. In contrast, exposure to 100 [51] and 800 [55] mg BPA kg^−1^ d.m. of soil increased AlP activity by 23% and 8%, respectively. Interestingly, similar to urease, the activation of this enzyme was due to the presence of hydroxyl and carboxyl groups configured in the phenyl ring, which enhances AlP adsorption to soil colloids with a positive effect [60].

It should also be emphasized that the soil pH is a major determinant of biochemical activity [61]. In particular, this parameter notably moderates not only enzyme sorption but also proteolysis and enzyme inactivation [62]. Moeskops et al. [63] postulate that an increase in pH affects the destabilization of ionic and hydrogen bonds in the active center of dehydrogenases. Conversely, Kappaun et al. [64] report that the optimal pH for urease is 7–8. Importantly, the synthesis of this enzyme by microorganisms may be their response to stress, including excessively low soil pH.

Of the seven sorbents verified for their bioremediation potential, two were selected: the molecular sieve and grass compost, which is particularly important for improving the biochemical properties of the soil. The compost revealed effectiveness against Deh, Cat, Ure, AcP, and AlP. The research results obtained are in line with the attributed function of this organic substance as a moderator in the C, N, and P cycles, closely associated with the stimulation of dehydrogenases, urease, and phosphatases [42,64]. Additionally, compost is recognized as a source of electrons essential for the catabolic neutralization or elimination of organic pollutants, including BPA, conducted through redox reactions [42]. The molecular sieve demonstrated efficacy against Deh, Cat, and Ure. Its capabilities are attributed to the high number of nanopores providing adsorption of pollutants, associated with the silicate hydroxyl group, specifically ion exchange on its surface [65]. Pre-fermented bark also attenuated the inhibitory effect of BPA on Glu activity and enhanced AlP activity. Its beneficial effect is probably due to the richness and diversity of polyphenolic structures, including phenolic acids, stilbenes, flavonoids, and glucosides [44].

### 3.2. Zea mays

The demonstrated sensitivity of *Zea mays* to increasing soil contamination with bisphenol (BPA) undoubtedly requires discussion and justification, taking into account the disturbance of root growth and development as well as the aerial parts of the plant. A much higher toxicity of BPA to *Zea mays* roots was observed in our research, similar to previous studies by other authors [47,66]. They also explain many of the mechanisms activated in response to this xenobiotic. One of the fundamental mechanisms is the reduction in the activity of nitrate reductase, which is responsible for the reduction of NO_3_^–^ to NO_2_^–^ in the cytosol of maize roots. Importantly, in response to BPA pressure, the activity of key enzymes involved in ammonia assimilation also decreases, occurring through the GS/GOGAT (glutamine synthetase/glutamine oxoglutarate aminotransferase) pathway and GDH—glutamate dehydrogenase [66].

The research results obtained can be justified by referring to the findings of Bahmani et al. [67], which indicate that BPA inhibits root growth by redistributing, or more precisely accumulating, auxin in the root meristem in the elongation zone. This process, in turn, is associated with increased expression of the PIN1 and PIN4 genes and decreased expression of the EXPA8 and 10 genes.

Another significant phenomenon that supports the greater toxicity of BPA to plant roots than to their aerial parts is the documented reduction in abscisic acid (ABA) content and increase in gibberellic acid (GA) and ethylene in roots, as reported by Li et al. [68]. The greater sensitivity of *Zea mays* roots compared to aerial parts to the applied xenobiotic in our studies may also be attributed to the increase in mitochondrial reactive oxygen species (ROS), including H_2_O_2_ and O_2_, as indicated by Xiao et al. [69], which are associated with cell death in the apical meristem of roots. According to the researchers, such drastic effects of BPA action are also evidenced by the increase in malondialdehyde (MDA) levels, a flagship marker of oxidative stress that reflects the degree of cell damage [70]. Growth and development disturbances in *Zea mays* could also result from a decrease in glutathione content in maize root cells, induced by an increase in glutathione peroxidase (GPX) and ascorbate peroxidase (APX) activity [71]. It is worth noting that Zhao et al. [66] demonstrated that exposure to 50 mg BPA kg^−1^ of soil reduced proline and protein content in maize seedling roots by 12% and 29%, respectively.

Based on the results of the research carried out, a significant trend observed was the induction of negative changes by BPA in the aerial parts of *Zea mays*, although this was not as pronounced as in the case of the roots. Undoubtedly, the hydrophobicity of BPA (log_Kow_ BPA = 3.40) plays an important role in the conditions obtained, favoring the weak migration of this phenolic compound, which consequently corresponds to lower bioconcentration factors of BPA in stems and leaves compared to plant roots [72]. One of the reasons for this is that the hydroxyl group of BPA interacts with binary ions in the soil, with which it forms conjugated estrogens, which in turn interfere with the mobility of this xenobiotic in the plant [73].

To mitigate excessive damage, including oxidative stress, plants activate defense systems against the adverse effects of bisphenols. As contested by Tossounian et al. [74], glutathione S-transferases (GSTs) play a very important role in the detoxification of exogenous and endogenous compounds in plants. The marker gene commonly used to assess plant sensitivity to stress induced by BPA is the enzyme ATGSTT8, assigned to the phi class of GSTs. An adaptive response to BPA toxicity also involves an increased accumulation of amino acids in the plant, such as arginine, serine, and tyrosine [75]. One mechanism that has been well-described by researchers [76,77] is the regulation of detoxification gene expression. This mechanism is particularly significant in *Zea mays*, as it corresponds to the activation of up to ten genes, including lignin, flavonoid, and phenylpropanoid genes. They are thought to have the function of initiating the phenylpropanoid pathway, providing a milder response of maize to BPA pressure [77].

An important research step was to determine the chlorophyll content in *Zea mays* leaves exposed to BPA, expressed as SPAD index values. The research results obtained were quite controversial, as many researchers [78,79,80] pointed to a disruption of chlorophyll synthesis in plant leaves, mediated by photosystem I (PSI) and photosystem II (PSII), mainly argued by the limitation of the function of the stomatal apparatus due to its reduced size and oxidative damage to photosynthetic pigments. However, in the current scientific discussion, it is necessary to take into account the fact that plants respond differently to BPA due to their species diversity and directly related to it, the different rates of BPA metabolism [81,82]. This hypothesis is confirmed by our previous research findings [56] in which exposure to 1000 BPA kg^−1^ d.m. of soil did not disrupt chlorophyll synthesis in the leaves of both *Zea mays* and *Brassica naupus*. The importance of this postulate may also be strengthened by the aforementioned hydrophobicity of the characterized phenolic compound, which determines its low mobility in the plant.

The starting point for analyzing the potential of bioremediating substances to mitigate the toxic effects of BPA on the growth and development of *Zea mays* was the fact that carbon assimilation by the plant results in the activation of reaction centers in the photosynthetic system, and that BPA inhibits this process [83]. The hypothesis put forward by the researchers explains the high SPAD values obtained, and thus the higher yield of *Zea mays* in plots with a combination of BPA and compost, as well as BPA and pre-fermented bark in our research. Both compounds significantly increased soil organic carbon content. In turn, the values of this parameter were correlated with the values of the C:N ratio.

## 4. Materials and Methods

### 4.1. Materials

#### 4.1.1. Soil

The soil for the study was collected from the morainic region of the undulating ground of the Olsztyn Lakeland, covering an area of 1845 km^2^. It was an agriculturally utilized area located at the geographical coordinates: NE Poland, 53.713° N, 20.432° E, in the northeastern part of Lakeland. The research was conducted on the soil of the Eutric Cambisol type (FAO 2006), sampled from a cultivated field at a depth of 0–20 cm. Before setting up the experiment in the vegetation hall of the Didactic-Experimental Centre of the University of Warmia and Mazury in Olsztyn, analyses were performed to characterize the basic properties of the tested soil using standard analytical methods. The particle size distribution was determined using the Malvern Mastersizer 3000 laser diffraction analyzer (Malvern, Worcestershire, UK) [84,85], revealing it to be loamy sand composed of sand (37.14%), silt (54.71%), and clay (8.15%). C_org_ and N_total_ contents were also determined and were 6.28 g and 1.16 g kg^−1^ of soil dry matter (d.m.), respectively, with a C:N ratio of 5.42. Hydrolytic acidity (HAC) was determined using the Kappen method (−17.25 mM(^+^) kg^−1^ d.m. of soil), along with exchangeable base cations (EBC)—196.00 mM(^+^) kg^−1^ d.m. of soil [86]. Based on the obtained values of HAC and EBC, two other soil properties were calculated: cation exchange capacity (CEC) (213.23 mM(^+^) kg^−1^ d.m. of soil), and alkaline cation saturation (ACS) (91.91%). The studied soil exhibited a neutral reaction (pH 6.7 in 1 mol KCl dm^−3^). pH determination was carried out using a pH meter HI 2221 (Hanna Instruments, Washington, DC, USA) [87]. The characterization of the soil was expanded to include its biochemical properties (Table 4).

#### 4.1.2. BPA

According to the Sigma Aldrich (St. Louis, MO, USA) Material Safety Data Sheet, bisphenol A (BPA) CAS: 80-05-7 is a crystalline white substance with a purity of ≥98.0% (HPLC). Its synonyms include 4,4′-isopropylidenediphenol and 2,2-bis(4-hydroxyphenyl)-propane). The most important physicochemical properties of BPA, which influence the interactions of this phenolic compound in soil, are the bioconcentration factor (BCF), vapor pressure (V_P_), and water solubility (S_W_) [91]. The fate of BPA in soil is significantly influenced by its hydrophobic properties [92]. These properties are also defined by the soil adsorption coefficient (log*K*_OC_) (Table 5) [26].

#### 4.1.3. Characteristics of Remediating Substances

In the experiment, the potentials of three mineral sorbents and three organic nutrient substances were identified. The group of mineral sorbents includes:

**Molecular sieve (M).** The average micropore diameter of the utilized aluminosilicate was 0.3 mm, with a pH of 8.5. According to the product specification, the maximum volatile content in the product at 950 °C was 2.5%. The commercial name of the product is Silosiv A3 (Company Sylosiv, Columbia, MD, USA).

**Zeolite Bio.Zeo.S.01 (Z).** The main compounds composing the zeolite used in the study are SiO_2_ and Al_2_O_3_, constituting 70.6% and 12.32%, respectively. The total content of Fe, Ti, Mn, Ca, Mg, K, and Na was 8.64%. The mineral applied is a source of clinoptilolite, which accounts for 60% of the zeolite. Clinoptilolite is characterized by a high cation exchange capacity and is used in zeoponic substrates for plant cultivation and as a fertilizer. The manufacturer of zeolite BIO.Zeo.S.1 is the company Bio-Drain (Rzeszów, Poland). It is an inorganic compound with a three-dimensional crystalline structure.

**Sepiolite (Sep).** A clay mineral (Mg_4_[Si_6_O_15_(OH)_2_]6H_2_O) produced by the company Sepiolsa Minersa Group (Guadalajara, Spain). According to the manufacturer, the product contained 70% sepiolite, with a pH_KCl_ = 7.1. Four key characteristics describing sepiolite include fibrous morphology, atypical pore structure, high adsorption capacity, and specific surface area. A comprehensive characterization of molecular sieve, sepiolite, and zeolite is presented in the research by Strachel et al. [93] and Boros et al. [94].

The three organic nutrient substances used are starch, grass compost, and pre-fermented bark.

**Starch (St).** According to the Sigma Aldrich data sheet, the molar mass of soluble starch (C_6_H_10_O_5_) is 162.1 g mol^−1^ and the water solubility is 50 g dm^−3^ (90 °C), with a pH range of 6.0–7.5.

**Compost (Cp).** The grass compost used in the experiment was characterized by the following parameters: N_total_ (20.18), C_org_ (146.61), soil organic matter (SOM, 252.76 g kg^−1^ d.m.), P (3.41), K (9.25), and Mg (5.69, mg kg^−1^ d.m.). The pH_KCl_ of the compost was 6.1. A detailed description of this nutrient can be found in the study by Wyszkowska et al. [95].

**Pre-fermented bark (Bk).** The supplier of pre-fermented bark from coniferous trees was the company “Athena Bio-Produkty” Sp. z o.o. (Golczewo, Poland). According to the manufacturer’s description, the product’s fraction size ranged from 20–50 mm, organic matter content ≥ 50%, dry matter content ≥ 30%, and pH_H2O_ ≤ 6.0.

#### 4.1.4. Characteristics of Zea Mays

*Zea mays* was first classified as a domestic crop in Mexico around 7000 BCE [96]. According to the FAO Report, the global maize harvest in 2023 increased by 1.2% (33.3 × 10^9^) compared to 2022, with global production of this plant exceeding 1 × 10^9^ annually (1147.7 × 10^9^) [97]. Maximizing the production and productivity of *Zea mays* meets the nutritional needs of the growing world population [98]. It is cultivated in more than 170 countries worldwide, covering approximately 193.7 million hectares. Maize has also attained the status of a global industrial crop, being used in 83% of the feed, starch, and biofuel industries [97]. In the experiment, the soil was sown with the maize of the DS1897B variety.

### 4.2. Methods

#### 4.2.1. Design and Procedure for Conducting a Greenhouse Experiment Using *Zea mays*

After conducting research aimed at determining the physicochemical and biochemical properties of soil sieved through a 0.5 cm mesh sieve, a pot experiment was set up in a greenhouse, with four replicates. The experimental factors were: (1) the level of soil contamination with BPA: 0, 500, 1000 mg BPA kg^−1^ d.m. of soil—3 objects—and (2) the type of remediation substance: control (Ct), molecular sieve (M), zeolite SO1 (Z), sepiolite (Sep), starch (St), compost from grass (Cp), pre-fermented bark (Bk)—7 objects—giving a total of 21 objects. As the experiment was conducted in four replicates, a total of 286 kg of soil packed at 3.4 kg in each of the 84 pots was used. Remediation substances were added to the individual treatments at a rate of 20 g kg^−1^ d.m. of soil. As the soil was sown with *Zea mays* to assess the sensitivity of this crop to the increasing pressure from the phenolic compound, pre-sowing fertilization with N and P (at 150 mg kg^−1^), K (at 50 mg kg^−1^), and Mg (at 20 mg kg^−1^) was necessary to provide optimum nutrient levels for maize. After thorough mixing with the soil, the xenobiotic (BPA), mineral fertilizers, and remediation substances, the soil material was packed into the pots, arranged in planned configurations, and the soil moisture level was adjusted to 60% of the maximum soil moisture content in each pot. Subsequently, eight maize (*Zea mays* L.) seeds were sown in each pot. Five days after sowing, when the coleoptile emerged on the soil surface, four plants were left in each pot. The experiment was terminated after 55 days by harvesting *Zea mays* and collecting soil samples for biochemical analyses as well as the determination of C_org_ and N_total_ content.

#### 4.2.2. Measurement of SPAD and *Zea mays* Biomass

According to the BBCH scale used in the European Union to identify the phenological stages of crops, plant harvesting was performed at the initial stage of the emergence of the panicle (BBCH 51). Just before harvesting, the average SPAD value was determined based on eight readings on the 5th leaf of each plant. The SPAD 502 Chlorophyll Meter 2900P (KONICA MINOLTA, Inc., Chiyoda, Japan) was used for measurements. On the day of harvest, the yield of aerial and root parts of *Zea mays* was estimated. The dry weight of maize was determined after 5 days of drying at 60 °C.

#### 4.2.3. Determination of Soil Enzyme Activities

To diagnose the condition of soil subjected to BPA pressure, the activity of seven soil enzymes was determined: dehydrogenases (Deh) [88] and catalase (Cat) [89], urease (Ure), acid phosphatase (AcP), alkaline phosphatase (AlP), *β*-glucosidase (Glu), and arylsulfatase (Aryl) [90]. The selection of these soil enzymes was dictated by the aim of obtaining holistic data on the extent of disruption of the biochemical transformations of carbon (Deh, Cat, Glu), nitrogen (Ure), phosphorus (AcP, AlP), and sulfur (Aryl), in which individual enzymes are involved. The significant parameters of individual biochemical analyses were as follows:

**Substrates**: Deh—2,3,5–Triphenyl tetrazolium chloride (TTC); Cat—H_2_O_2_—aqueous solution; Ure—urea—aqueous solution; AcP and AlP—Disodium 4-nitrophenyl phosphate hexahydrate (PNP); Glu—4-nitrophenyl-*β*-d-glucopyranoside (PNG); Aryl—Potassium-4-nitrophenylsulfate (PNS),

**Products**: Deh—triphenyl formazan (TFF); Cat—O_2_; Ure—N-NH_4_; AcP, AlP, Glu, and Aryl-4-nitrophenol (PN). The activity of Deh, Ure, Glu, Aryl, AcP, and AlP was determined, in triplicate, using a Perkin-Elmer Lambda 25 spectrophotometer (Waltham, MA, USA). A detailed description of the methods used to determine the activity of all enzymes has been provided in previous studies [99].

#### 4.2.4. Methodology for Calculations and Statistical Data Analysis

The scale of the inhibitory interaction of BPA was verified using the bisphenol influence factor (IF_BPA_). The potential of the applied mineral sorbents and organic nutrient substances to mitigate the hypothetical negative effect of BPA on enzyme activity, as well as on the growth and development of *Zea mays*, was traced through the prism of the influence factor of the remediation substance (IF_Rs_) on the studied parameters. The response of the seven enzymes was verified and described based on the BPA influence index (IF_BPA_) and the index of bioremediating substances (IF_R_s) on the parameters tested. The formulas used to determine the index values are:

(1) $${IF}_{BPA}=\frac{A_{BPA}}{A_{C}}-1$$

where:

IF_BPA_—index of influence of soil contamination with BPA

IF_Rs_—index of influence of bioremediating substances

IF_BPA_Rs_—<1—inhibition; >1—stimulation of individual enzyme activity and growth and development of *Zea mays*

A_BPA_Rs_—activity of individual enzymes and yield of *Zea mays* in soil contaminated with 500 and 1000 mg BPA kg^−1^ d.m. of soil

A_C_—activity of individual enzymes and yield of *Zea mays* in soil not contaminated with BPA

(2) $${IF}_{Rs}=\frac{A_{Rs}}{A_{C}}-1$$

where

IF_Rs_—index of influence of bioremediating substances

IF_Rs_—<1—inhibition; >1—stimulation of individual enzyme activity and growth and development of *Zea mays*

A_Rs_—activity of individual enzymes and yield of *Zea mays* in soil contaminated with 500 and 1000 mg BPA kg^−1^ d.m. of soil

A_C_—activity of individual enzymes and yield of *Zea mays* in soil not contaminated with BPA

Based on the sum of the activities of seven enzymes assigned to each treatment, the biochemical soil fertility index (BA_21_) was also calculated, the formula for which was proposed in the publication by Wyszkowska et al. [100]. Additionally, the ratio of aerial parts biomass to root biomass was calculated [56]. To verify the obtained data, an analysis of variance (ANOVA) was conducted to describe the percentage of variability of the analyzed variable (η^2^). Principal component analysis (PCA) was used to trace the interdependencies between thirteen parameters, including seven enzymes (Deh, Cat, AcP, AlP, Ure, Glu, Aryl), nitrogen content (N_total_), organic carbon content (C_org_), the leaf greenness index (SPAD) of *Zea mays*, aerial parts biomass (Ya), root biomass (Yr), and soil pH. Tukey’s test at *p* = 0.05 was used to determine homogeneous variances between the variables [101].

## 5. Conclusions

Bisphenol A proved to be a significant moderator of soil biochemical activity. Application of this phenolic compound to soil had a negative effect on the activity of all enzymes analyzed, except alkaline phosphatase and catalase. However, the crux of the response to the research question regarding the extent of BPA inhibition highlighted the particular sensitivity of dehydrogenases, urease, and acid phosphatase to this xenobiotic, regardless of the level of contamination applied. Soil contamination with 500 mg BPA kg^−^^1^ d.m. of soil resulted in an 82% inhibition of Deh activity, while 1000 mg kg^−^^1^ d.m. of soil generated an 89% inhibition compared to the control. In contrast, exposure to increasing doses of this xenobiotic resulted in 55% and 94% inhibition of Ure activity, respectively. As enzymes serve as early indicators of soil equilibrium disturbances, their response to BPA was also reflected in the growth and development of *Zea mays*. Toxicological stress caused a dramatic inhibition of the root system growth and consequently a reduction in the aerial parts biomass of the cultivated plant. Soil contamination at 500 and 1000 mg BPA kg^−^^1^ d.m. of soil reduced aerial part yield by 44% and 71% and Zea mays root yield by 60% and 84%, respectively. Therefore, the use of remediation substances to verify their potential was highly justified. The application of compost from grass and molecular sieves can be recommended as an effective practice to be implemented within common agricultural practices. They not only neutralize the toxic effects of BPA on soil enzymes but also restore the soil’s ability to promote *Zea mays*. The results obtained can be described as a matrix for further research steps in the search for substances that not only restore soil health but also reduce the sensitivity of *Zea mays*, a plant used for energy production, to the effects of BPA.

## Figures and Tables

**Table 1:** Enzyme activity in 1 kg d.m. of soil per 1 h.

Object	0_BPA	500_BPA	1000_BPA	Average
Dehydrogenases, µmol TFF				
Control (Ct)	10.636 ^b^	1.955 ^f–h^	1.167 ^h^	4.586 ^C^
Molecular sieve (M)	13.484 ^a^	6.509 ^c^	7.051 ^c^	9.015 ^A^
Zeolite (Z)	10.079 ^b^	1.038 ^h^	1.379 ^gh^	4.165 ^D^
Sepiolite (Sep)	10.754 ^b^	3.366 ^de^	2.285 ^e–g^	5.468 ^B^
Starch (St)	10.355 ^b^	2.674 ^ef^	2.413 ^e–g^	5.147 ^B^
Compost (Cp)	10.754 ^b^	4.160 ^d^	1.094 ^h^	5.336 ^B^
Bark (Bk)	10.012 ^b^	1.863 ^f–h^	1.358 ^gh^	4.412 ^C^
Average	10.868 ^I^	3.081 ^II^	2.392 ^III^	
Catalase, mol O_2_				
Control (Ct)	0.243 ^g^	0.238 ^g^	0.274 ^f^	0.252 ^D^
Molecular sieve (M)	0.305 ^de^	0.343 ^c^	0.335 ^c^	0.328 ^B^
Zeolite (Z)	0.313 ^d^	0.159 ^i^	0.179 ^h^	0.217 ^E^
Sepiolite (Sep)	0.334 ^c^	0.180 ^h^	0.272 ^f^	0.262 ^CD^
Starch (St)	0.340 ^c^	0.239 ^g^	0.237 ^g^	0.272 ^C^
Compost (Cp)	0.387 ^a^	0.287 ^ef^	0.368 ^b^	0.347 ^A^
Bark (Bk)	0.287 ^ef^	0.339 ^c^	0.366 ^b^	0.331 ^B^
Average	0.316 ^I^	0.255 ^III^	0.290 ^II^	
Urease, mmol N-NH_4_				
Control (Ct)	0.345 ^g^	0.156 ^i^	0.020 ^j^	0.174 ^F^
Molecular sieve (M)	1.512 ^b^	0.430 ^f^	0.252 ^h^	0.731 ^B^
Zeolite (Z)	1.211 ^d^	0.028 ^j^	0.026 ^j^	0.422 ^E^
Sepiolite (Sep)	1.329 ^c^	0.030 ^j^	0.114 ^i^	0.491 ^D^
Starch (St)	1.685 ^a^	0.351 ^g^	0.311 ^g^	0.783 ^A^
Compost (Cp)	1.206 ^d^	0.225 ^h^	0.246 ^h^	0.559 ^C^
Bark (Bk)	1.132 ^e^	0.252 ^h^	0.132 _i_	0.505 ^D^
Average	1.203 ^I^	0.210 ^II^	0.157 ^III^	
Acid phosphatase, mmol PN				
Control (Ct)	4.058 ^c^	2.456 ^fg^	2.364 ^gh^	2.959 ^C^
Molecular sieve (M)	2.319 ^gh^	1.915 ^i^	1.411 ^j^	1.882 ^E^
Zeolite (Z)	1.499 ^j^	2.663 ^ef^	2.321 ^gh^	2.161 ^D^
Sepiolite (Sep)	2.773 ^e^	2.118 ^hi^	1.554 ^j^	2.148 ^D^
Starch (St)	5.990^a^	2.674 ^ef^	2.250 ^gh^	3.638 ^B^
Compost (Cp)	5.249 ^b^	3.494 ^d^	3.511 ^d^	4.085 ^A^
Bark (Bk)	4.267 ^c^	1.031 ^k^	0.943 ^k^	2.081 ^D^
Average	3.737 ^I^	2.336 ^II^	2.051 ^III^	
Alkaline phosphatase, mmol PN				
Control (Ct)	0.330 ^m^	0.558 ^j–l^	0.544 ^j–l^	0.477 ^F^
Molecular sieve (M)	0.525 ^j–l^	0.605 ^h–j^	0.487 ^kl^	0.539 ^E^
Zeolite (Z)	0.460 ^l^	0.518 ^j–l^	0.579 ^i–k^	0.519 ^E^
Sepiolite (Sep)	0.751 ^fg^	0.803 ^ef^	0.877 ^de^	0.810 ^C^
Starch (St)	0.738 ^fg^	0.556 ^j–l^	0.686 ^gh^	0.660 ^D^
Compost (Cp)	0.978 ^cd^	0.667 ^g–i^	0.984 ^c^	0.876 ^B^
Bark (Bk)	0.719 ^fg^	1.458 ^a^	1.229 ^b^	1.135 ^A^
Average	0.643 ^II^	0.738 ^I^	0.769 ^I^	
Arylsulfatase, mmol PN				
Control (Ct)	0.176 ^b–d^	0.141 ^e–g^	0.121 ^gh^	0.146 ^C^
Molecular sieve (M)	0.177 ^bc^	0.156 ^c–f^	0.179 ^bc^	0.171 ^B^
Zeolite (Z)	0.197 ^b^	0.097 ^h^	0.093 ^h^	0.129 ^D^
Sepiolite (Sep)	0.171 ^b–d^	0.233 ^a^	0.169 ^b–e^	0.185 ^A^
Starch (St)	0.228 ^a^	0.148 ^d–g^	0.135 ^f–g^	0.170 ^B^
Compost (Cp)	0.192 ^b^	0.141 ^e–g^	0.148 ^d–g^	0.158 ^C^
Bark (Bk)	0.163 ^c–f^	0.162 ^c–f^	0.156 ^c–f^	0.160 ^BC^
Average	0.186 ^I^	0.154 ^II^	0.143 ^III^	
β-glucosidase, mmol PN				
Control (Ct)	0.472 ^de^	0.377 ^h–k^	0.357 ^i–k^	0.402 ^C^
Molecular sieve (M)	0.435 ^e–h^	0.345 ^jk^	0.260 ^l^	0.346 ^D^
Zeolite (Z)	0.417 ^e–i^	0.467 ^d–f^	0.331 ^k^	0.405 ^C^
Sepiolite (Sep)	0.425 ^e–h^	0.381 ^g–k^	0.350 ^jk^	0.384 ^C^
Starch (St)	0.808 ^a^	0.344 ^jk^	0.405 ^f–j^	0.519 ^B^
Compost (Cp)	0.568 ^c^	0.447 ^e–g^	0.553 ^c^	0.522 ^B^
Bark (Bk)	0.569 ^c^	0.732 ^b^	0.519 ^cd^	0.606 ^A^
Average	0.528	0.442 ^II^	0.396 ^III^	
	BA_21_			
Control (Ct)	16.260 ^c^	5.880 ^g–i^	4.848 ^jk^	8.996 ^E^
Molecular sieve (M)	18.757 ^b^	10.303 ^e^	9.977 ^e^	13.012 ^A^
Zeolite (Z)	14.175 ^d^	4.970 ^jk^	4.909 ^jk^	8.018 ^F^
Sepiolite (Sep)	16.536 ^c^	7.111 ^f^	5.620 ^i–k^	9.756 ^D^
Starch (St)	20.145 ^a^	6.986 ^fg^	6.437 ^f–i^	11.189 ^C^
Compost (Cp)	19.333 ^ab^	9.422 ^e^	6.903 ^f–h^	11.884 ^B^
Bark (Bk)	17.148 ^c^	5.836 ^h–j^	4.703 ^k^	9.229 ^F^
Average	17.478 ^I^	7.216 ^II^	6.200 ^III^	

BPA—bisphenol A; 0_BPA, 500_BPA, 1000_BPA—doses of BPA kg^−1^ d.m. of soil. Homogeneous groups were determined individually for each enzyme and BA_21_ (represented by letters ^a–m^); averaged values, regardless of the level of soil pollution with BPA, are marked with letters ^A–F^, while the mean values, regardless of the type of remediation substance, are indicated by numbers ^I–III^. significant at *p* = 0.05, *n* = 63.

**Table 2:** The yield of *Zea mays* (d.m. g pot^−1^).

Object	0_BPA	500_BPA	1000_BPA	Average
Aerial parts				
Control (Ct)	46.475 ^bc^	26.130 ^g^	13.570 ^l^	28.725 ^B^
Molecular sieve (M)	48.129 ^bc^	31.109 ^f^	25.732 ^g^	34.990 ^A^
Zeolite (Z)	45.988 ^c^	20.702 ^h^	15.896 ^jk^	27.529 ^BC^
Sepiolite (Sep)	44.333 ^d^	24.965 ^g^	15.868 ^ij^	27.066 ^B^
Starch (St)	35.693 ^d^	23.165 ^h^	15.246 ^jk^	24.559 ^C^
Compost (Cp)	52.997 ^a^	32.924 ^f^	17.999 ^kl^	34.640 ^A^
Bark (Bk)	48.352 ^b^	20.419 ^i^	9.400 ^m^	26.057 ^BC^
Average	45.995 ^I^	25.632 ^II^	16.244 ^III^	
Roots				
Control (Ct)	11.813 ^a^	4.771 ^d^	1.937 ^fg^	6.174 ^AB^
Molecular sieve (M)	11.581 ^a^	3.909 ^de^	2.757 ^ef^	6.082 ^AB^
Zeolite (Z)	11.795 ^a^	4.520 ^d^	2.409 ^fg^	6.241 ^A^
Sepiolite (Sep)	8.700 ^b^	4.864 ^d^	2.382 ^fg^	5.315 ^B^
Starch (St)	11.281 ^a^	5.054 ^d^	2.950 ^ef^	6.428 ^A^
Compost (Cp)	11.218 ^a^	6.368 ^c^	3.171 ^ef^	6.919 ^A^
Bark (Bk)	12.166 ^a^	5.150 ^cd^	1.137 ^g^	6.151 ^AB^
Average	11.222 ^I^	4.984 ^II^	2.392 ^III^	

BPA—bisphenol A; 0_BPA, 500_BPA, 1000_BPA—doses of BPA kg^−1^ d.m. of soil. Homogeneous groups were determined individually for each part of *Zea mays* (represented by letters ^a–m^); averaged values, regardless of the level of soil contamination with BPA, are marked with letters ^A–C^, while the mean values, regardless of the type of remediation substance, are indicated by numbers ^I–III^. significant at *p* = 0.05, *n* = 63.

**Table 3:** The mean values of SPAD on the 50th day of growth of *Zea mays*.

Object	0_BPA	500_BPA	1000_BPA	Average
Control (Ct)	35.835 ^ij^	37.946 ^f–i^	41.066 ^c–e^	39.618 ^ABC^
Molecular sieve (M)	36.277 ^h–j^	39.610 ^d–g^	38.960 ^e–h^	38.842 ^C^
Zeolite (Z)	34.803 ^j^	38.952 ^e–h^	41.351 ^c–e^	39.957 ^ABC^
Sepiolite (Sep)	38.025 ^f–i^	42.337 ^cd^	41.600 ^c–e^	41.407 ^AB^
Starch (St)	21.226 ^l^	35.797 ^ij^	39.884 ^d–f^	36.676 ^BC^
Compost (Cp)	36.843 ^g–j^	47.983 ^a^	43.646 ^bc^	44.095 ^A^
Bark (Bk)	30.439 ^k^	45.873 ^ab^	40.655 ^de^	40.969 ^BC^
Average	33.350 ^II^	41.214 ^I^	41.023 ^I^	

BPA—bisphenol A; 0_BPA, 500_BPA, 1000_BPA—doses of BPA kg^−1^ d.m. of soil. Homogeneous groups were determined for all objects (represented by letters ^a–l^); averaged values, regardless of the level of soil pollution with BPA, are marked with letters ^A–C^, while the mean values, regardless of the type of remediation substance, are indicated by numbers ^I,II^, significant at *p* = 0.05, *n* = 63.

**Table 4:** Some biochemical properties of the soil used in the experiment.

* Type of Enzyme	Enzymatic Activity per 1 kg d.m. h^−1^	Unit	Methodical References
Deh	3.716	µmol TPF	[88]
Cat	0.210	mol O_2_	[89]
Ure	0.266	mmol N-NH_4_	[90]
AcP	3.207	mmol PN	
AlP	0.712	mmol PN	
Aryl	0.132	mmol PN	
Glu	0.381	mmol PN	

* Ure—urease, AcP—acid phosphatase, AlP—alkaline phosphatase, Aryl—arylsulfatase, Glu—*β*-glucosidase, TPF—triphenyl formazan, PN—4-nitrophenol.

**Table 5:** Selected physicochemical properties of BPA [89].

Acronym	Molecular Weight g mol^−1^	Total Formula	Structural Formula	BCF	log*K*_OC_	*S*_W_ mg dm^−3^	*V*_P_ (Pa)
BPA	228.29	C_15_H_16_O_2_	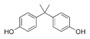	71.85	4.88	120	5.6 × 10^−6^

## Data Availability

Data are available by contacting the authors.

## Abbreviations

BPA—bisphenol A, 0, 500, 1000—doses of BPA kg^−1^ d.m. of soil, Ct—soil without remediating substances, M—molecular sieve, Z—zeolite, Sep—sepiolite, St—starch, Cp—grass compost, Bk—fermented-bark, Deh—dehydrogenases, Cat—catalase, Ure—urease, AcP—acid phosphatase, AlP—alkaline phosphatase, Glu—*β*-glucosidase, Aryl—arylsulfatase, BA_21_—the biochemical fertility index, IF_BPA_–index of the influence of BPA, IF_RS_—index of the influence of remediating substances, SPAD—the leaf greenness index, PR—the ratio of the mass of the aerial parts of plants to the roots, Y_a_—aerial part of plants, Y_r_—roots of plants, N_total_—total nitrogen, C_org_—organic carbon.
